# Development of Older Adult Food Insecurity Index to Assess Food Insecurity of Older Adults

**DOI:** 10.1007/s12603-022-1816-6

**Published:** 2022-07-05

**Authors:** Jenny Jin Young Lee, S. Shen, C. Nishita

**Affiliations:** 1 Thompson School of Social Work and Public Health, University of Hawai'i at Mānoa, Honolulu, HI, USA; 2 Department of Urban and Regional Planning, University of Hawai'i at Mānoa, Honolulu, HI, USA

**Keywords:** Measurement, nutrition, food insecurity, risk factors, GIS

## Abstract

**Objectives:**

Quantifying the number of older adults that are food insecure in a specific geographic area is critical in developing and scaling public health prevention and response programs at the local level. However, current estimates of older adult food insecurity only consider financial constraints, following the same methodology as the general population, even though the drivers for older adults are different and multidimensional. This study aims to build a general approach to quantify the food-insecurity among older adults at the local level, using publicly available data that can be easily obtained across the country.

**Methods:**

13 risk factors for food insecurity among older adults were identified leveraging existing studies, following the Social Ecological Model (SEM), and the weighted impact of each factor was determined. Publicly available data sources were identified for each factor, ZIP code level data was compared to national averages, and the weighted data for each factor were aggregated to determine the overall food insecurity at the local level.

**Results:**

Based on the averaged odds ratios across all the studies, of the 13 risk factors, beyond financial constraints, having a disability was the most impactful factor and distance to the nearest grocery store was the least impactful. A ZIP code level model of Honolulu County was developed as an example to demonstrate the approach, showing that food insecurity among older adults in the county was 2.5 times that which was reported from the Current Population Survey (16.5% versus 6.5%).

**Conclusion:**

This evidence-based model considered factors that impact food insecurity among older adults across all the spheres of the SEM. The drivers of food insecurity among older adults are different than the drivers for the general population, resulting in a higher percentage of older adults being food insecure than currently reported.

## Introduction

**T**he Current Population Survey (CPS) identified that more than 5.3 million older adults in the U.S. were food insecure as of 2018 (1). However, according to existing studies on the topic, this likely understates the actual prevalence of food insecurity for this population segment. The CPS uses the USDA Household Food Security Survey Module (HFSSM) to identify food insecure older adults (2). The HFSSM mainly captures food access issues resulting from financial resource constraints, which are the primary drivers for such insecurity in the general population (3, 4, 5). However, older adults experience food insecurity differently from other age groups. For instance, anxiety related to food access is a more significant aspect of food insecurity among older adults (6, 7). Physical and cognitive challenges, as well as many comorbidities, are additional important factors connected to food insecurity among older adults (8, 9, 10, 11); however, the CPS does not take these factors into account (3, 4, 5, 12). Food insecurity rates are also higher among older adults who live alone, are socially isolated, or experience functional limitations (13).

The importance of incorporating additional factors beyond financial constraints to measure food insecurity among older adults has been discussed in numerous studies. For example, Vilar-Compte et al. reviewed 58 existing studies related to food insecurity among older adults (14). They identified that food insecurity among older adults is significantly associated with “age, race and ethnicity, marital status, gender, health status, depression, functionality, income, poverty, household composition and homeownership” (14). Given these additional factors that need to be considered when estimating food insecurity rates for older adults, Wolfe et al. proposed additional measures as supplements to the HFSSM (4). They emphasized that the HFSSM only looks at financial constraints; however, older adults can be food insecure even if they have enough money to purchase food due to functional impairments that limit their ability to access, prepare or eat appropriate meals. As such, they created a 14-question supplement to the HFSSM that better captured food insecurity for older adults and compared the results of this survey to the standard HFSSM. As an example, one of the additional questions was, “I worried whether my food would run out because I couldn't get the food I needed even though I had money for food.” This was asked in addition to the HFSSM question, “I worried whether my food would run out before I had money to buy more.” By comparing the results of the two surveys, they found that more than twice the number of older adults were food insecure based on their new measurement versus the ones identified using the HFSSM (4). While the sample size of this study was small (46 households), it provided insight into the likelihood that the estimated number of older adults that are food insecure is significantly higher than that reported in the CPS.

The advent of the COVID-19 pandemic further increased the percentage of older adults that were food insecure, which is also not captured in the traditional survey-based HFSSM approach. Existing meal programs were impacted by the shutdown of congregate meal sites, and home-delivered meal programs were disrupted owing to a shortage of volunteer drivers, as the majority of drivers were older individuals themselves. Additionally, older adults were fearful of going out to shop for groceries due to the fear of infection, limiting their access to fresh groceries. Support networks were also disrupted due to job loss and restrictions on visits to prevent the spread of the virus. Finally, financial resource constraints worsened for some older adults due to job loss and increased medical expenses. Schanzenbach and Northwestern University identified that the impacts of COVID-19 exacerbated food insecurity in 2020, as nearly 60% more older adults are food insecure compared to 2018 (15).

While numerous studies identify the impact of factors associated with food insecurity among older adults, and in some cases build models that predict food insecurity based on these factors, they rely on data that is not available at a refined local geographic level (i.e., at a county, ZIP code, or Census tract level) (5, 12, 16). While some research presented localized assessment models, these models could only be applied to specific locales, such as a certain State or foreign nation, due to their intensive requirements for comprehensive data (17, 18, 19, 20). A general model that can predict the number of food-insecure older adults at the local level and can be applied generally across the nation is needed, so that service organizations at different geographical levels can appropriately scale to better identify the vunerable population, support their needs, compare between regions, allocate fundings accordingly, and be prepared to recover from a public health emergency quickly.

Previous studies investigated factors associated with food insecurity among older adults through secondary data analysis and in-depth interviews (5, 12, 13, 16, 17, 20, 21, 22, 23, 24). A number of the studies leveraged the Social Ecological Model (SEM) as a conceptual framework to explore the factors associated with food insecurity among older adults since it provides a systematic multidimensional approach to investigating the issue (5, 12, 25). Goldberg and Mawn, using the SEM, revealed the antecedents of food insecurity within the older population at a national level by leveraging the National Health and Nutrition Examination Survey (12). Building on Goldberg and Mawn's research, Tucher et al. employed the SEM to establish a summary indicator of food insecurity specific to older adults through the National Health and Aging Trends Study (5). Through their study, Tucher et al. highlighted that food insecurity among older adults is associated with social and functional limitations in addition to financial constraints. However, the current measure of food insecurity among older adults only considers financial constraints and does not capture other important factors that impact food insecurity among older adults. In addition, no literature identifies a model that can predict older adult food insecurity at a local geographic level and be easily generalized nationwide.

As such, the purpose of this study was a) to apply the SEM model to examine a comprehensive set of factors that might impact food insecurity and b) to develop a model, called the Older Adult Food Insecurity Index (OAFII), to provide a better estimate of older adults that are food insecure, at a local level but can also be generalized using existing data across the nation, to act as a foundation for improved nutrition support and emergency response programs.

## Method

To develop the OAFII, it was necessary to 1) identify risk factors associated with food insecurity among older adults within the SEM based on existing literature, 2) determine which of these factors could be quantified with publicly available data (Census, Food Access Research Atlas) at a sufficiently granular geographic level, 3) weight each factor to quantify their relative influence on food insecurity based on existing literature, 4) determine the weighted impact of the geographic data relative to the national average for each factor, 5) aggregate the weighted data for the factors (other than financial resources) to determine the relative non-financial impact, and finally, 6) combine the impact of these additional factors with food insecurity data identified based on just the financial drivers to determine the overall older adult food insecurity at a local level.

### Identify Factors and Data Availability

The first step in creating the OAFII was identifying factors associated with food insecurity among older adults. First, a comprehensive database search of existing literature was conducted, including four databases (CINAHL, PychINFO, PubMed, Web of Science) using the following search terms:

• # 1 “elderly” [Title/Abstract] OR «aging»[Title/Abstract] OR «aged»[Title/Abstract])
• # 2 «food insecurity»[Title/Abstract] OR «hunger»[Title/Abstract] OR «malnutrition» [Title/Abstract]
• # 3 «poverty»[Title/Abstract] OR «socio ecological model»[Title/Abstract] OR «social isolation» [Title/Abstract] OR «depression»[Title/Abstract] OR «cognition»[Title/Abstract] OR «mobility limitation»[Title/Abstract] OR «disability»[Title/Abstract]
• # 4 #1 AND #2 AND #3

The search was conducted in November 2021 and was not limited to peer reviewed jounal articles. After identifying qualified studies, reference lists from selected studies were then manually searched to identify additional studies. Inclusion criteria was research with target group age 65+, related to risk factors associated with food insecurity among older adults, conducted since 1999, and written in English. Exclusion criteria included research focusing on all age groups as well as health outcomes or risk factors unrelated to food and nutrition. As a result, 18 existing studies that addressed the factors associated with food insecurity among older adults were identified (4, 5, 12, 13, 14, 16, 17, 18, 19, 20, 22, 23, 24, 26, 27, 28, 29, 30).

Leveraging the SEM lens, the factors associated with older adult food insecurity included factors in all five spheres (intrapersonal, interpersonal, institutional, community, and policy/social factor). These included policy/social factors (receipt of nutritional assistance, Medicaid, income assistance), community factors (climate, neighborhood characteristics, urban/rural environments, congregate meals sites), institutional factors (distance to the grocery store, ability to cook meals, health insurance, access to routine healthcare), interpersonal factors (emotional and financial support, social isolation, community disability), and intrapersonal factors (poverty level, gender, age, race/ethnicity, length of time in the U.S., marital status, education level, being homebound, IADLs, ADLs, BMI, and depression) (5, 14, 31).

**Figure 1:**
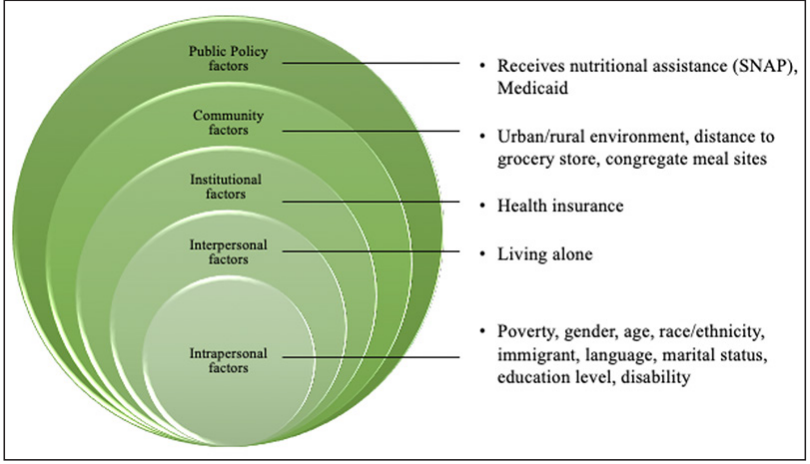
Factors with publicly available data in the Social Ecological Model

The second step was to determine whether there was publicly available data at a sufficiently detailed local level for each factor. Identifying easily accessible data is crucial as it helps generalize the application of such index at different geographical scales (e.g., county, ZIP code, or even Census tract level). Leveraging data from the Census American Community Survey (ACS) (32) and the Food Access Research Atlas (33), sufficient details for 16 variables were identified to be incorporated into the OAFII (Figure 1).

### Weighting of Factors and Geographic-Level Data

A detailed review of the 18 studies was conducted to identify information on the relative impact of each factor associated with food insecurity among older adults. Of the 18 studies used to determine the factors to incorporate into the model, nine studies were excluded from the analysis of the relative impact due to lack of data, such as small sample size, different metric definitions, or lacking connection to publicly available data (4, 13, 14, 26, 27, 28, 29, 30, 34). From the remaining nine studies, odds ratios were determined for factors associated with food insecurity for older adults. In those studies, data was available, either based on bivariate or multivariate analysis conducted by the authors, to determine the odds ratio associated with one or more factors. For example, if the rate of food insecurity for women was twice the rate of food insecurity for men, then the odds ratio for food insecurity for women would be 2.0 relative to men. Then, since the odds ratios were different between studies (based on the data used or factors investigated), to determine the odds ratio for each factor, we averaged the odds ratios across all the studies that show a statistical significance for the particular factor:

$$W_{a}=\frac{\sum_{i=1}^{n}{OR}_{ai}}{n}$$

*W*_*a*_: *National weighting for factor “a”*
*OR*_*ai*_: *Odds Ratio for factor “a” from study “i”*

Of the 16 factors initially identified, three factors were excluded through the process of determining their weighting impact in the model. Distance to congregate meal sites was removed since the only study that specifically identified an odds ratio for this factor determined that it was not statistically significant (12). Percent foreign born and primary language other than English were identified as factors but none of the studies that identified these as drivers of older adult food insecurity contained sufficient data to determine odds ratios. The 13 remaining factors were percent of 65+ below poverty, female, being younger age (age 65 to 74), race other than white, not married, educational level less than high school graduate, disability (Intrapersonal factors); living alone (Interpersonal); without health insurance (Institutional factors); living in an urban setting, at least ½ mile from grocery store (community factors); on Medicaid, and below poverty but not receiving SNAP (policy/social factors).

Percent of 65+ below poverty was used as a proxy for financial constraints associated with food insecurity, allowing current food insecurity data to be determined at a more detailed geographic level. For the twelve remaining factors, excluding financial drivers, the relative impact of the factors on older adult food insecurity were primarily determined based on national level data (33 of the 52 odds ratios incorporated were based on U.S. national level data, with another 10 coming from U.K. or Canadian national level data). Therefore, to understand the relative impact of the data for any specific geography, it was necessary to compare the geographic-level data with the national average for that factor and then weigh that factor.

The geographic-level data for each factor is based on percentiles of the population (for example, percent of population 65+ female, which could range from 0% to 100%). As such, it wasn't necessary to normalize the data for each factor.

### Aggregation of Impact

To create a national impact metric, the 12 weighted non-financial factors at the national level were multiplied by each other. While the relative impact of each individual factor could be determined from the odds ratios in the previously mentioned nine studies, the combined impacts of the 12 non-financial factors relative to financial constraints were only discussed in a single study. Wolfe et al. estimated that the actual number of food-insecure older adults was double the number currently identified by the HFSSM, which is based purely on financial constraints (4). As such, this model values the national impact of the combined 12 non-financial factors as a 100% increase relative to the food insecurity level determined by the HFSSM.

$$INF=I_{1}*I_{2}*\ldots *I_{n}$$

*INF*: *National non-financial factor impact*
*I*_*a*_: *Impact of factor “a”*

This approach of multiplying odds ratios ensures that, if all other factors were held constant, then the odds ratio for the remaining factor would determine the difference in food insecurity (as long as the underlying data was indexed from 0 to 1). This makes the assumption that the factors are all independent of each other. This is unlikely to be the case (for example, since women live longer than men on average, the higher the percent female in the older adult population, it is likely that there will be a lower percent of the population under 75); however, this was taken into account by using the odds ratios from studies that developed multivariate models where available.

This same process could then be completed for each specific geography. The weighted data for each factor were multiplied by each other and then divided by the national average impact to determine the relative impact for each geography. This relative geographic impact was then multiplied by the national impact of the non-financial factors on food insecurity rates to determine the effect in each geography.

$$NFFI_{b}=\left(\frac{I_{b1}*I_{b2}*\ldots *I_{bn}}{INF}\right)*NFFI$$

*NFFI*_*b*_: *Non-financial food insecurity rate for geography “b”*
*I*_*bi*_: *Impact for factor “i” in geography “b”*
*INF*: *National non-financial factor impact*
*NFFI*: *National non-financial food insecurity rate (determined leveraging CPS and Wolfe et al.)* (4)

Finally, the food insecurity determined from financial constraints (from the HFSSM and using percent below poverty as a proxy to determine relative geographic differences) and the impact of the aggregated non-financial factors were combined to determine the OAFII for each geography:

$$FII_{b}=(NFFI_{b}+(\frac{I_{Pb}}{WA_{Pc}}*FFI_{c}))/FFI$$

*FII*_*b*_: *Food Insecurity Index for geography “b”*
*NFFI*_*b*_: *Non-financial food insecurity rate for geography “b”*
*I*_*pb*_: *Impact of poverty “P” in geography “b”*
*WA*_*Pc*_: *Weighted Average impact of poverty “P” in broader geographical analysis area “c”*
*FFI*_*c*_: *Financial food insecurity rate for geography “c”*
*FFI*: *National financial food insecurity rate, from CPS*

## Results

**Figure 2:**
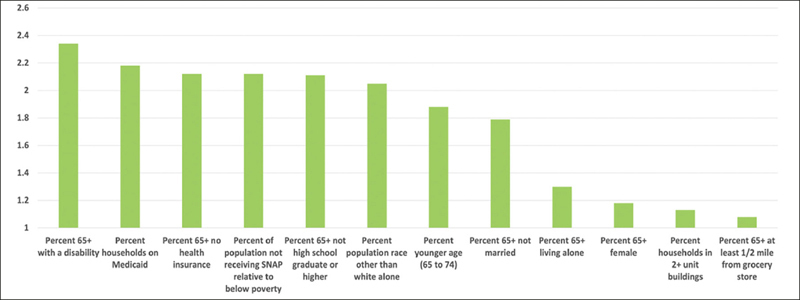
Weighting of Non-Financial Risk Factors

The odds ratios for the 12 non-financial factors that were included in the final version of the model ranged from most impactful (having a disability, such as hearing, vision, cognitive, ambulatory, self-care, and independent living difficulty (35), with a weighting of 2.34) to least impactful (percent at least a ½ mile from a grocery store, with a weighting of 1.08) (Figure 2).

The model approach was tested at county, ZIP code, and Census tract levels for multiple geographies. To illustrate the detailed model findings, a ZIP code level analysis of Honolulu County is incorporated as an example. The non-financial factor impacts are summarized in Table 1. One ZIP code area, Kunia (96759), was removed from the final analysis since it was determined to be an outlier due to the very small sample size of older adults.

**Table 1:** Impact of non-financial risk factors for Honolulu County

**ZIP Codes**	**Percent female over 65+**	**Percent 65+ under 75years old**	**Percent of population race other than white alone**	**Percent 65+ currently not married**	**Percent 65+ not high school graduate or higher**	**Percent 65+ with a disability**	**Percent of 65+ living alone**	**Percent 65+ without health insurance**	**Percent of households in 2+ unit buildings**	**Percent of seniors at least 1/2 mile from grocery store**	**Percent of population not receiving SNAP relative to below poverty**
96701	1.10	1.48	1.93	1.31	1.09	1.43	1.04	1.00	1.05	1.05	1.56
96706	1.10	1.55	1.88	1.36	1.27	1.52	1.03	1.01	1.03	1.06	1.55
96707	1.10	1.50	1.85	1.36	1.17	1.46	1.05	1.00	1.04	1.06	1.38
96712	1.09	1.63	1.40	1.37	1.12	1.43	1.05	1.00	1.01	1.06	1.57
96717	1.09	1.54	1.78	1.35	1.20	1.55	1.06	1.02	1.03	1.07	1.68
96730	1.08	1.66	1.68	1.42	1.16	1.43	1.10	1.00	1.02	1.08	1.65
96731	1.10	1.55	1.72	1.33	1.15	1.30	1.05	1.02	1.02	1.05	1.69
96734	1.10	1.51	1.53	1.32	1.06	1.42	1.05	1.01	1.02	1.05	1.85
96744	1.10	1.44	1.82	1.33	1.10	1.45	1.05	1.00	1.03	1.05	1.67
96762	1.11	1.70	1.69	1.25	1.04	1.26	1.03	1.00	1.02	1.02	1.77
96782	1.10	1.44	1.93	1.31	1.11	1.44	1.04	1.00	1.03	1.05	1.68
96786	1.10	1.40	1.73	1.38	1.21	1.49	1.06	1.01	1.04	1.05	1.71
96789	1.10	1.54	1.89	1.28	1.10	1.41	1.05	1.00	1.05	1.04	1.78
96791	1.10	1.55	1.61	1.33	1.25	1.47	1.05	1.01	1.03	1.06	1.92
96792	1.09	1.55	1.94	1.40	1.20	1.60	1.04	1.01	1.04	1.06	1.69
96795	1.10	1.48	1.95	1.43	1.19	1.56	1.03	1.01	1.02	1.06	1.74
96797	1.11	1.47	1.98	1.34	1.28	1.50	1.03	1.00	1.05	1.03	1.69
96813	1.10	1.50	1.88	1.44	1.20	1.45	1.10	1.00	1.10	1.04	1.50
96814	1.11	1.48	1.91	1.47	1.11	1.39	1.14	1.00	1.12	1.00	1.62
96815	1.09	1.57	1.65	1.42	1.10	1.36	1.12	1.01	1.11	1.02	1.92
96816	1.11	1.46	1.84	1.39	1.11	1.42	1.06	1.00	1.03	1.05	1.86
96817	1.10	1.44	1.96	1.39	1.34	1.41	1.07	1.00	1.08	1.02	1.68
96818	1.10	1.46	1.75	1.34	1.21	1.42	1.04	1.00	1.05	1.04	1.85
96819	1.11	1.47	1.99	1.40	1.30	1.47	1.03	1.01	1.04	1.05	1.74
96821	1.10	1.42	1.82	1.32	1.05	1.40	1.06	1.00	1.01	1.07	1.92
96822	1.10	1.44	1.83	1.38	1.08	1.41	1.08	1.00	1.09	1.04	1.87
96825	1.10	1.47	1.74	1.26	1.03	1.33	1.05	1.00	1.03	1.07	1.88
96826	1.10	1.47	1.88	1.51	1.22	1.46	1.13	1.00	1.12	1.00	1.76
County	1.10	1.48	1.85	1.36	1.17	1.44	1.06	1.00	1.05	1.04	1.73

The OAFII is an index of older adult food insecurity rates relative to the rate of older adult food insecurity identified by the CPS at the national level. The higher the values are, the percent of food insecure older adult population in that geography is greater. As such, the index could range in value from 0 (meaning that there is no older adult food insecurity in that particular geography) to 13.7 (meaning that 100% of the older adult population in a particular geography is food insecure, given that the current national rate of food insecurity from the CPS is 7.3% and 100%/7.3% = 13,7).

**Figure 3:**
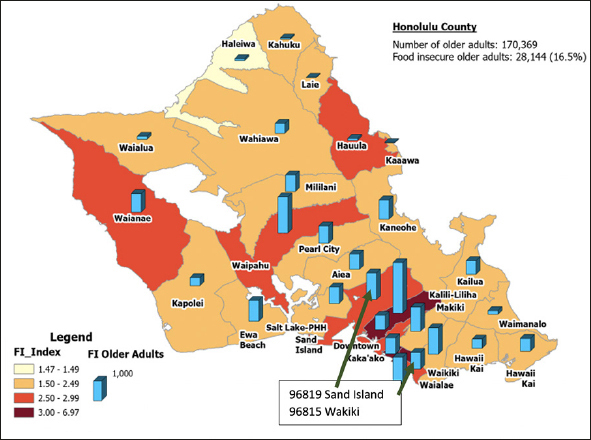
Map of Older Adult Food Insecurity Index for Honolulu County at a ZIP code level

The OAFII results for Honolulu County (Table 2) were developed by combining the impact of the non-financial drivers of food insecurity with the level of food insecurity due to financial constraints. The ZIP code area with the highest financial constraint index was McCully-Mo'ili'ili (96826) at 2.33, and the lowest was Hawaii Kai (96821) at 0.38. The ZIP code area with the highest non-financial factor index was McCully-Mo'ili'ili (96826) at 1.78, and the lowest was Haleiwa (96712) at 0.93. For the overall index (OAFII), the ZIP code with the highest index was McCully-Mo'ili'ili (96826) at 3.92, and the lowest was Haleiwa (96712) at 1.42. This information is also presented in map form in Figure 3.

**Table 2:** Older Adult Food Insecurity Index for Honolulu County

**ZIP Codes**	**Additional factors**	**Food insecurity from additional factors**	**Financial drivers of food insecurity**	**Food insecurity from financialdrivers**	**Overall Index**	**Food Insecurity Rate**
96701	1.15	0.08	0.63	0.04	1.72	13%
96706	1.15	0.11	0.80	0.05	2.18	16%
96707	1.09	0.08	0.54	0.04	1.59	12%
96712	0.93	0.07	0.53	0.04	1.42	10%
96717	1.43	0.10	1.37	0.09	2.68	20%
96730	1.42	0.10	0.88	0.06	2.22	16%
96731	1.15	0.08	1.02	0.07	2.09	15%
96734	1.04	0.08	0.53	0.04	1.53	11%
96744	1.13	0.08	0.48	0.03	1.57	11%
96762	1.03	0.07	0.70	0.05	1.67	12%
96782	1.22	0.09	0.59	0.04	1.76	13%
96786	1.30	0.09	0.85	0.06	2.08	15%
96789	1.26	0.09	0.44	0.03	1.67	12%
96791	1.61	0.12	0.69	0.05	2.25	16%
96792	1.65	0.12	1.17	0.08	2.73	20%
96795	1.60	0.12	0.52	0.03	2.07	15%
96797	1.53	0.11	1.15	0.08	2.58	19%
96813	1.41	0.10	1.41	0.09	2.70	20%
96814	1.41	0.10	2.07	0.14	3.31	24%
96815	1.44	0.10	1.49	0.10	2.81	20%
96816	1.38	0.10	0.70	0.05	2.03	15%
96817	1.62	0.12	2.06	0.14	3.51	26%
96818	1.34	0.10	1.01	0.07	2.27	17%
96819	1.77	0.13	1.00	0.07	2.68	20%
96821	1.21	0.09	0.38	0.03	1.56	11%
96822	1.45	0.11	0.70	0.05	2.10	15%
96825	1.05	0.08	0.63	0.04	1.63	12%
96826	1.78	0.13	2.33	0.16	3.92	29%

To explain further what this data highlights, one can compare two ZIP codes in Table 2 with relatively similar rates of older adult food insecurity. For ZIP code 96819 (Sand Island), the OAFII model predicts that older adult food insecurity is 2.68 times the current national average from the HFSSM data (with 19.6% of older adults living in the area being food insecure). Of these food-insecure older adults, 6.7% are food insecure due to financial constraints and 12.9% are food insecure due to other factors (i.e., non-financial drivers of food insecurity are almost twice as impactful as financial drivers in this ZIP code). For ZIP code 96815 (Waikiki), older adult food insecurity is similar to ZIP code 96819 (2.81 times the current national average, with 20.5% of older adults being food insecure). However, the drivers of food insecurity in ZIP code 96815 are very different than ZIP code 96819 (with food insecurity rates due to non-financial and financial constraints being almost the same at 10.5% and 10.0%, respectively). This variance between ZIP codes is due to the differences in demographics where one area (96819) has a higher percentage of older adults that are minorities, are less likely to have completed high school, and more likely to be on Medicaid, but a lower percentage of these older adults are living below the poverty line. These factors have a relatively higher impact on food insecurity than the other non-financial factors (see Figure 2). As such, it is critical to look at non-financial drivers in addition to financial drivers to determine the rate of food insecurity among older adults in a given area.

## Conclusions

In the modern era, social scientists have become more informed and sophisticated about how to provide goods and services to vulnerable older populations. In recent times we have learned of the importance of culturally competent services and ethically appropriate foods. We now know that communal or congregate meals are vital for socialization, and human contact and social networking. Evidence has further informed us that home delivered meals are a lifeline to older persons who are not mobile, and that the volunteer or staff delivering that meal may be the only human contact the older adult may encounter for days at a time. The recent COVID pandemic has exposed the deadly impact of isolation and loneliness on all people, but older adults in particular.

This study developed a model that provided a better estimate of the number of older adults that are food insecure by incorporating the specific non-financial constraints into consideration in the assessment. The drivers of food insecurity among older adults are multidimensional and require a broader approach to determine the level of food insecurity in this vulnerable population group. This evidence-based model provided a comprehensive approach that incorporated factors that impact food insecurity among older adults across all the spheres of the SEM. By utilizing readily accessible data that can be obtained across the nation, the approach provides a general framework that is easily adaptable to different regions and geographical scales. It fills a gap in assessing the drivers and predictors of food insecurity among older adults at the local level. The approach could provide support to develop more targeted and efficient strategies to address social and public health problem that need better and more sophisticated explanations and predictions than the national average estimate. It could help to more efficiently allocate the limited resources to populations and geographic locations that are more at risk.

An evidence-based model better-predicting food insecurity also informs social science/public health curricula, city, county and agency budgets and public policy. Because this approach is regionally scalable and easily customizable at the local level, government agencies and non-profit organizations might utilize it to assist in planning and scaling food security initiatives. For example, if a county were developing a new meal program, this model could help them understand the number of food-insecure older adults at a census tract level, so that they could prioritize key geographic response areas and ensure sufficient scale of response efforts. This model could also be used to optimize existing programs by determining which geographic areas were receiving good coverage and where additional resources were needed, by comparing actual results with predicted need. Leveraging this same information, existing providers could seek additional funding to meet the identified gaps relative to demand, and funders would have confidence that resources were going to the areas with the greatest need.

The multidimensional nature of older adult food insecurity, and its association with multiple negative health outcomes, also means that clinicians have to address the consequences, often without recognizing that this is the underlying cause (36). Through understanding the drivers and the prevalence within a specific service area, clinical personnel can prioritize screening for food insecurity and referrals to appropriate services that can help maintain health and extend independent living (37). By assessing older adults for food insecurity, health care providers can help overcome stigmas, tailor clinical care to real patient needs, and potentially reduce health care costs by reducing preventable emergency visits and hospitalizations (38).

Additionally, GIS and mapping are increasingly used to bridge public health research with neighborhood-level information in multiple disciplines. The detailed geographic level data used in the development of this model allows identifying the number of older adults that are food insecure at a Census tract, ZIP code, or county level. Combining this OAFII model with a GIS data mapping tool (Figure 3) provides visualization of where resources are most needed and can be used by response organizations to identify areas of greatest need and compare their program efforts at a detailed geographic level to identify areas needing additional resources and where there is potential overlap. This information can then be used to develop support and intervention programs, provide a foundation for funding and policy creation, and serve as an initial estimate for emergency response initiatives. The ability to measure older adult food insecurity reliably is increasingly vital in resource management for planning intervention strategies and responding to public health emergencies, and the Older Adult Food Insecurity Index developed through this study provides a widely available tool for organizations to use in this effort.

One of the limitations of this predictive model is that it relies on the limited number of published studies on older adult food insecurity that quantify the impact of non-financial factors. In particular, this research only discovered one previous study that examined the impact of non-financial factors relative to the impact of financial constraints (4). Understanding the importance of factors beyond financial constraints and the specific impact of those factors relative to financial constraints is critical in developing and scaling food insecurity prevention and response programs, given that the current measurement approach significantly understates the number of older adults impacted by food insecurity. To increase the validity and accuracy of this model, further research on the impact of non-financial drivers of older adult food insecurity should be conducted to validate such understanding. Additionally, the predictive accuracy of the model would benefit from the implementation of detailed comparison research, leveraging the supplemental older adult food insecurity survey developed by Wolfe et al., for a detailed set of geographies (4). This form of research could be used to both confirm and improve the validity of this model (4).
